# Liquid and Solid Functional Bio-Based Coatings

**DOI:** 10.3390/polym13213640

**Published:** 2021-10-22

**Authors:** Vito Gigante, Luca Panariello, Maria-Beatrice Coltelli, Serena Danti, Kudirat Abidemi Obisesan, Ahdi Hadrich, Andreas Staebler, Serena Chierici, Ilaria Canesi, Andrea Lazzeri, Patrizia Cinelli

**Affiliations:** 1 Department of Civil and Industrial Engineering, University of Pisa, 56122 Pisa, Italy; vito.gigante@dici.unipi.it (V.G.); luca.panariello@ing.unipi.it (L.P.); serena.danti@unipi.it (S.D.); andrea.lazzeri@unipi.it (A.L.); 2 Interuniversity Consortium of Materials Science and Technology (INSTM), 50121 Florence, Italy; 3 IRIS Technology Solutions (IRIS), 08940 Cornellà de Llobregat, Spain; kabidemi@iris-eng.com; 4 Biomass Valorization Platform-Materials, CELABOR s.c.r.l., 4650 Chaineux, Belgium; ahdi.hadrich@celabor.be; 5 Fraunhofer-Institute for Process Engineering and Packaging, 85354 Freising, Germany; andreas.staebler@ivv.fraunhofer.de; 6 Stazione Sperimentale per l’Industria delle Conserve Alimentari (SSICA), 43121 Parma, Italy; serena.chierici@ssica.it; 7 Planet Bioplastics s.r.l., 56017 Pisa, Italy; ilariacanesi@planetbioplastics.it

**Keywords:** coatings, active molecules, barrier properties

## Abstract

The development of new bio-based coating materials to be applied on cellulosic and plastic based substrates, with improved performances compared to currently available products and at the same time with improved sustainable end of life options, is a challenge of our times. Enabling cellulose or bioplastics with proper functional coatings, based on biopolymer and functional materials deriving from agro-food waste streams, will improve their performance, allowing them to effectively replace fossil products in the personal care, tableware and food packaging sectors. To achieve these challenging objectives some molecules can be used in wet or solid coating formulations, e.g., cutin as a hydrophobic water- and grease-repellent coating, polysaccharides such as chitosan-chitin as an antimicrobial coating, and proteins as a gas barrier. This review collects the available knowledge on functional coatings with a focus on the raw materials used and methods of dispersion/application. It considers, in addition, the correlation with the desired final properties of the applied coatings, thus discussing their potential.

## 1. Introduction

The production of items derived from sustainable and renewable resources, not toxic for humans and the environment, is a pressing challenge facing our society [1]. In this context, the production of sustainable coatings with improved and multifunctional performances is necessary [2]. As such, the search for coatings that have to be bio-based, with good barrier, water resistance and antimicrobial features is underway [3]. Nowadays, extensively used materials, with excellent moisture barrier properties for the production of coatings, are fundamentally petro-based. This must be the barrier to break down in research in the coming years [4]. 

**Figure 1:**
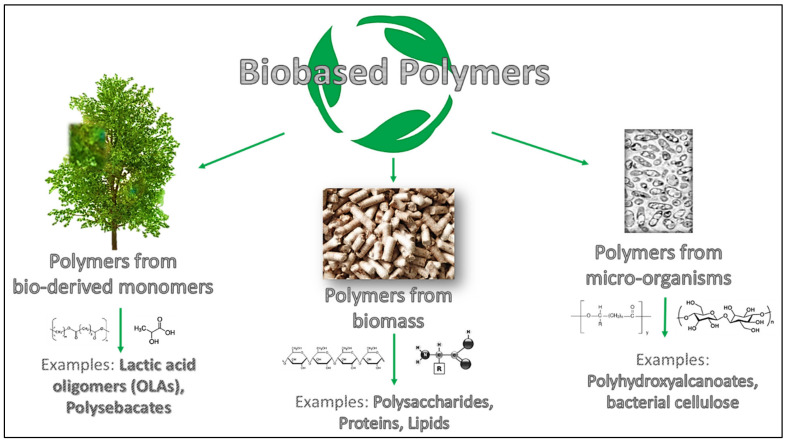
Schematic overview of bio-based polymers’ differences.

Before going into the detail of the review subject, it is necessary to underline and to clarify the definition of biodegradable and bio-based polymers used for coatings formulation. The concept of biodegradation refers to biodegradable polymers that can be disintegrated and catabolized to CO_2_ and H_2_O by bacteria and/or enzymes [5,6]. Instead, bio-based polymers can be categorized based on their source, process technique, and formulation following the classification shown in Figure 1 [7]. In detail, bio-based polymers can be derived from biomass (like polysaccharides, lipids and proteins), from bio-derived monomers achieved by fermentation, such as lactic acid oligomers (OLAs); finally it is possible to find polymers developed from microorganisms (e.g., polyhydroxyalkanoates, PHAs) [8]. 

In the field of coatings, these bio-based polymers represent the “new pathway to follow” because they can specifically provide to substrates multiple functionalities, also in relation to their processing conditions, without being petro-based [9]. 

Generally, functional bio-based coatings can be applied with the aim to improve the surface characteristics of a substrate (adhesion, wettability, water repellence, anti-corrosion properties and gas barrier. In other cases the coating can guarantee new properties in the final product, being an essential part of it [10].

This review will, therefore, be focused on the state of the art of bio-based and sustainable coatings production, with a detailed analysis of their application on cellulosic and plastic substrates. Moreover, the involvement of biomolecules in the coating formulations, but also the main technological innovations and the difference among liquid and solid preparation of bio-based sustainable coatings will be described in the following sections.

Indeed, to develop sustainable coatings for cellulose or bioplastic substrates is a technological goal of huge importance and it has become mandatory in the bioeconomy and circular economy context, aimed at imparting proper functional characteristics, based on biopolymer and functional materials coming from agro-food waste streams [11]. 

Coatings based on polymers, polymeric composites, and nanocomposites are used in several applications and sectors: (aerospace, automotive, marine structures, biomedical devices, decorative stuff, energy items, packaging). High-quality material is usually attained by thoroughly modulating layer/substrate.

Starting from paper substrates, it is well known that they are made of the most available bio-based material: the cellulose [12]. The use of cellulose-coated materials in personal care and disposable items for food (i.e., tableware) could be helpful for the environment and it is a route that has been followed in recent years [13]. The limits yet to be overcome are the hydrophilicity and low barrier properties typical of a non-woven fibrous system. For this reason, materials based on cellulose combined with poly(ethylene) are still widely present on the market, and these petro-based products are currently preferred, despite their negative environmental impact. 

Regarding the application of coatings on plastic substrates, this is becoming increasingly necessary with the development of items with novel bio-based and biodegradable plastics based for example on poly (lactic acid) PLA, poly (butylene succinate) PBS, poly(butylene succinate-adipate) PBSA, as they do not show adequate barrier properties and are not able to withstand the rigours of the market [14,15,16,17]. In fact, since they do not present barrier properties comparable to traditional plastics [18], they need a protective layer. Obviously, in order not to affect the renewability and biodegradability of the product, a coating must also be developed with the characteristics of being bio-based and environmentally friendly [19].

Therefore, a considerable research effort “is on the agenda” investigating and formulating new bioplastics and new sustainable coating systems [20]. While their actual impact on the market is growing, it needs to be sharpened in many other applications.

The critical issue to achieving real progress towards sustainable materials is to intercept society’s willingness to achieve sustainability; consumers must understand that obtaining sustainable products also means reducing global costs [21].

## 2. Bio-Based Coatings—Properties, Processing, Testing and Applications

### 2.1. Key Properties of Bio-Based Coatings

The innovation on bio-based coatings accompanies food packaging novelties and personal care applications. More specifically, the largest part of the bio-based coating research activity is primarily focused upon low-end (i.e., short-lived) bioplastic-based food packaging and paper coating for personal care. In contrast, fewer innovations are dedicated to coating for high-end (i.e., durable) applications [22]. Food products, indeed, endure many chemical, physical, and bacterial modifications when stored [23]. The shielding coating achieved during processing retards the damaging food deterioration, but also its quality is improved. For this reason, modification of the packaging, together with the development of eco-sustainable materials for packaging applications [24], can slow down deterioration rate of the packaged product, and hence, extend the shelf life of food [25]. 

Regarding personal care products, the goal to achieve is to reach tailored specific functional assets via a proper coating that can widen a large range of application, improving properties and favouring their use [26]. In addition, the production of bio-based films to coat personal care products, able to provide antimicrobial properties through the insertion of active biomolecules into a primer, is an encouraging alternative with respect to the direct application of antimicrobials in the food [27].

Nonetheless, to increase sustainability, the polymers should be bio-based, but green synthesis methods, which favour the use of non-toxic and environmentally friendly solvents, preferably relying on water-based or powder coatings, should be adopted [28]. Furthermore, coating cellulose or bioplastics with proper functional coatings based on biopolymer and functional materials deriving from agro-food waste streams will improve their performances, thus enabling them to replace effectively fossil-based products in personal care, tableware, and food-packaging sectors [29]. 

Table 1 briefly describes the most employed biopolymers used as a coating on cellulosic or plastic substrates, their preparation, application methods (that will be evidenced more n-depth in Section 4) and their key properties.

Connecting bio-based coating properties to final applications can be very useful in new product development. Those relations will be more extensively considered in the following sections of this review. In particular, three main properties for coatings will be considered:

- Antimicrobial coatings produced with chitin nano-fibrils and/or chitosan can be useful for cellulose tissues (e.g., personal care), paper and cardboard (e.g., packaging for fresh products like pasta, tableware), woven and non-woven (e.g., sanitary, personal care), plastic substrates (e.g., bio-polyesters) for active packaging.
- Gas barrier improvements for multilayer food packaging (e.g., bio polyester-based), with sustainable end of life options could be achieved by protein-based coatings
- Water-repellent properties for paper cups, but also non-food packaging, could be imparted by including cutin, thanks to its hydro-repellence

Polymeric coatings can be applied on several substrates, using many technologies, and with different approaches that depend on the nature of the coating (i.e., liquid or solid, detailed in the next sections). Although going in depth into the details of such technologies is behind the purpose of this review a marginal description of the main technologies, such as extrusion/dispersion coating and solution application, is necessary to comprehend how to exploit and develop bio-based coatings [40].

Thermoplastic polymers can be applied on bioplastic or cellulosic substrates with the technique of cast extrusion coating. Differently, biopolymers lacking of thermoplastic behaviour—as for example proteins, polysaccharides and fatty acids—can be also coated by polymer dissolution in a suitable solvent, or dispersing it in a solvent via dispersion coating [9].

Anyway, the use of these renewable materials in coatings faces issues and technical challenges due to low adhesion of the bio-based coatings on both plastic and cellulosic substrates [41]. Indeed proteins, chitosan and chitin have shown difficulty in adhering to plastic substrates; coating of cellulosic substrates have to face the challenge of moisture and temperature sensitivity [42].

### 2.2. Main Physico-Chemical Surface Treatments and Measurement Protocols

There are many physical and chemical processes employed for activating the surface of materials. Plasma-treated wood presented a substantially improved adhesion to the coating, leading to increased durability and a reduced attack by blue stain fungi. In the Durawood project [43], plasma was used as a pre-treatment before wood coating. Plasma-treated wood presented a substantially improved adhesion to the coating, leading to an increased durability, as well as a reduced attack by blue stain fungi. Unlike chemical treatment, plasma treatment does not require the use of chemicals and does not generate by-products. It can be promising for surface decontamination and finally for process intensification as it is expected to speed up the impregnation of the applied liquid.

Moreover, it is envisaged that coatings of several microns thickness will be applied to reach the multifunctional requirements of these applications, possibly in a subsequent step. As such, monitoring of these characteristics is needed. A number of monitoring techniques exist for thin printed coatings in the sub-micro/micro ranges. Most of them are in fact implemented off line and require sample preparation. 

Most of them are used offline and require sample preparation. However, according to a recent review article, some combined optical techniques have shown potential for this type of in situ analysis [44]. Spectral reflectance is the most frequently employed technology giving quantifiable data. A white light beam is directed onto the specimen surface and the reflectance is gathered and studied by a spectrometer. Thickness is computed by determining the wavelengths of the interference peaks in the reflectance spectrum, where the thickness of the layer is a function of the wavelength of the peak and the refractive index of the material. [45]. This method is ideal for a thickness between 1 and 50 microns.

As far as the testing methods are concerned, several protocols and procedures have been developed to test antimicrobial properties, gas and water barriers.

An interesting review has shown several methods to evaluate antimicrobial properties [46]. The official standards were published by the Clinical and Laboratory Standards Institute (CLSI) for bacteria and yeasts testing [47], being the agar disk-diffusion test the mainly used technique. In this procedure, microorganism were inoculated by agar plates following standard procedures. Then, filter paper discs are placed on the agar surface. The Petri dishes are protected under suitable conditions. Commonly, the antimicrobial agent diffuses into the agar and inhibits germination and growth of the tested microorganism and then the diameter of growth inhibition zone (i.e., called “halo”) is measured [48].

Regarding the barrier properties, the oxygen permeability, according to ASTM D3985-81, is evaluated as oxygen transmission rate (OTR) and demarcated as the oxygen amount passed through the material of a fixed thickness per unit of area and time [49]. 

The capacity of water vapour to permeate is measured, according to ASTM E96, instead, as water vapour transmission rate (WVTR), i.e., the quantity of water that passes through a substance of fixed thickness per unit of area and time [50]. The wettability or surface hydrophobicity can be evaluated through static or dynamic water contact angles [51]. Moreover, specifically for paper substrates, water absorption can be defined by the Cobb test (ISO 535). The Cobb value describes the water absorption capacity of a carton-board expressed in g/m^2^. If the COBB value is high, the substrate shows the ability to absorb and retain moisture, otherwise the substrate can withstand penetration and retention of moisture [52].

## 3. Innovative Coatings Based on Chitosan-Chitin, Proteins and Cutin

### 3.1. Innovation on Chitosan- and Chitin-Based Coatings

Coatings with antimicrobial agents are useful because they can protect surfaces to microbial growth and can also be employed as barriers to humidity and oxygen [53]. 

Among the biomolecules that can be helpful to guarantee antimicrobial properties, a lot of interest is focused on chitin (and its derivate: chitosan), which is also the second most abundant biopolymer on the earth with an annual production of 10^12^–10^14^ tons [54,55]. Speaking of numbers, the global demand for chitin in 2015 was above 60,000 tons, while its global production was around 28,000 tons [56]. Chitosan market size was valued at €1.5 billion in 2019, and is projected to reach €4 billion by 2027, according to a report by Global Industry Analysis [57]. The necessity of proper use of this waste material may allow the recovery of value-added goods also in the field of bio-based coatings. The amorphous part of chitin is transformed in chitosan by deacetylation. The difference between chitin and chitosan is not strict and it depends from the degree of deacetylation. Chitosan is a fully or partially deacetylated derivative of chitin, with a typical degree of deacetylation not higher than 65% [58]. Moreover, it can have animal (e.g., shells of crustaceans) or vegetal (e.g., fungi, such as *Aspergillus niger*) origin. Chitosan is characterized by non-toxicity, biodegradability, film-forming capacity, antimicrobial and antioxidant properties and good oxygen barrier properties [59]. The main advantage of chitosan application is the possibility to produce films and coatings with intrinsic antimicrobial properties which mainly differentiates chitosan from the other common antimicrobials (e.g., ethanol, sorbic acid, bacteriocins, lysozyme, essential oils) [60]. 

The properties of chitosan are related to origin and physico-chemical characteristics. Referring to films and coatings, antimicrobial and barrier properties depend on the molecular weight of chitosan, deacetylation degree, concentration, solvent used for its solubilisation, pH and possible plasticizers or other additives added in the formulations. The antimicrobial activity of chitosan relies on its positive charges, which can interact with negatively charged residues of macromolecules on the microbial cell surface, finally causing membrane leakage [61]. It is thus possible to find many examples of coatings, applied by dipping technique, spraying and other methods, as well as films produced by casting technique for fruit and vegetables, meat, cheese and fish, which avail themselves of chitosan. Antimicrobial properties of chitosan have been largely studied, even in combination with other substances, such as essential oils, or with other film-forming materials, such as proteins and gelatine. The use of chitosan for the edible coatings of fresh vegetables was investigated in depth recently by Tampucci et al. [62] who highlighted the possibility of developing a nutraceutical active coating for tomatoes. 

Interestingly, chitin nanofibrils (CNs) can be formed by controlling the deacetylation step, thus avoiding the full conversion to chitosan [63]. In fact, the CNs represent the crystalline part of chitin. The amorphous part of chitin is transformed in any case in chitosan by deacetylation.

CNs have attracted significant interest because of their peculiar properties, including exceptional mechanical properties (Elastic Modulus with values up to 140 GPa), thermal stability (around 300 °C), low density (≈1.5 g/cm^3^), renewable bio-based biodegradable and biocompatible character, biological properties, high aspect ratio and high surface area with a wide chemical modification capacity [64]. The first studies on CNs focused on their production processes by applying shear forces using mechanical treatment for physical disintegration of the cell wall along the longitudinal axis. The common mechanical treatments for the defibrillation of chitin fibres are based on high-pressure homogenizer and disk mills [65], less conventional ball milling [66], or high intensity ultrasonication [67]. However, thanks to the tough hydrogen bonds between chitin fibers, large quantities of energy are needed to their disintegration into nanofibers via mechanical treatments. To circumvent the problem of high energy consumption during the defibrillation processes, the mechanical treatment was combined with chemical pretreatment such as (2,2,6,6-tetramethylpiperidine-1-oxyl radical)-mediated oxidation (TEMPO) which was used to weaken the bonds that hold the chitin chains together, facilitating their conversion into CNs [68]. Partial deacetylation associated with partial mechanical scission of the fibrils during disintegration was also used to obtain CNs [69]. In addition, the esterification of hydroxyl groups of chitin by carboxylate groups can significantly improve the mechanical disintegration of chitin using a grinder [70]. Furthermore, a simple acidic treatment of chitin fibres coupled with mechanical treatment using grinder can accelerate their conversion into CNs thanks to the repulsive force caused by the cationization of amino groups [71]. Unfortunately, most of these methods require the use of toxic solvents, which significantly reduce the environmental benefits of CN [72]. 

Regarding the preparation of poly(lactic acid) (PLA)-based nanocomposites containing CNs, a fine dispersion was achieved thanks to the preparation of pre-mixtures, as described by Coltelli et al. [73,74]. This strategy can be considered to uniformly disperse CNs in biopolyester formulations or hot-melt oligopolyesters for producing functional film or coatings. CNs have been demonstrated to be cytocompatible, interestingly showing anti-inflammatory activity, which make them good vectors for the distribution of biomolecules for skin care and cells restoration [75]. All these findings are suggestive for promising applications in the personal care sector, because of the good compatibility of the CNs with the skin [76,77]. Recent studies are also considering CNs coatings and nanocomposites for some biomedical applications, such as eardrum repair [78].

### 3.2. Innovation on Protein-Based Coatings

As bio-based materials are potentially useful for protective coatings, the proteins play a fundamental role [79,80,81]. Specific advantages of proteins (easy to make into films and abundance) allow them to be used extensively for preparing biodegradable films [82].

Proteins are natural polymers synthesized by all living organisms for a wide range of reasons. There are twenty different monomeric units, called proteinogenic amino acids, whereas the structure and properties of a specific protein is determined by the number, sequence and types of amino acid. Therefore, different proteins as oxygen barrier layers have received some attention in the literature [83,84,85,86]. The excellent barrier properties of protein-based films are due to covalent and non-covalent intermolecular interactions caused by free functional groups of the amino acids in the polypeptide chain. These cause the formation of a protein network, acting as an efficient barrier for oxygen [87,88,89]. However, as a result of these interactions, protein-based films and coatings are usually brittle and require to be added with plasticisers [90]. Glycerol (GLY), a characteristic polyol, shows high capacity to resist to the water, and it can be added to the solution to increase the ductility of the final film [91]. On the other hand, these plasticisers increase oxygen permeability due to the increased free volume in the protein network [80]. Therefore, developing suitable protein-based formulations combining both good barrier as well as mechanical properties is of utmost importance [92]. 

Micellar proteins obtained from different sources have been used to develop a lacquering adhesive having the unique property of combining a high adhesive strength with an excellent barrier against oxygen [93]. Unfortunately, the adhesive strength could not be quantified as a rupture of the paper substrate occurred before the protein coating failed. This, however, indicates that the bond strength of the coating was exceeding the cohesion strength of the substrate [94]. Because of the huge capability to act against the oxygen permeation, protein-based polymers are helpful for producing sustainable coatings more than polysaccharides and lipids. For example, the oxygen permeability of soy protein-based films is lower with respect to pectin, starch and even polyethylene (PE) according to Schmid et al. [95]. Clearly, the tremendous gas barrier improvement and the increasing of mechanical resistance make the protein-based biopolymers one of the most useful solutions for the future trends in packaging [96]. 

### 3.3. Innovation on Cutin-Based Coatings

Cutin is a crosslinked polyester formed mainly by condensed polyhydroxylated acid [97] and is the main constituent of the cuticles of the plant. The primary role accredited to plant cuticles is to be water repellent, to avoid leakages from internal tissues [98,99]. They also act as gas obstacles, UV inhibitors and thermal controllers [100]. Cutin can be depolymerized by cleaving the ester bonds using alkaline hydrolysis, with NaOH or KOH in water, transesterification with methanol containing BF_3_ or NaOCH_3_, reductive cleavage by exhaustive treatment with LiAlH_4_ in THF, or with trimethylsilyl iodide in organic solvents [101]. Nevertheless, these methodologies are not satisfactory for large-scale cutin extraction, because of the steps involved and the impact of solvents and chemicals in terms of environmental and economic sustainability. Instead, the method patented by Cigognini et al. [102] is solvent-free and does not require pretreatment for cuticle isolation. This innovation allowed a pilot plant to be designed that extracts cutin from tomato by-products at a semi-industrial scale [103].

The first application of tomato cutin was the development of a bio-lacquer to coat food metal packaging. This application was patented and consists of a solvent based formulation [102]. Insoluble and thermostable coatings have been prepared from aleuritic acid as it is or added to palmitic acid, by melt-condensation polymerization in air without using solvents and catalysts [97,104]. The polyesters formulated can substitute plastic polymers or be applied as a coating. Tomato cutin was used in combination with sodium alginate and beeswax in a green solvent (i.e., water and ethanol) to obtain a hydrophobic free-standing film [105]. The work revealed that the thermal treatment (i.e., 150 °C, 8 h) represents a sustainable route to create structured, composite networks. Manrich et al. described the combination of cutin with pectin for the production of water-resistant plastic wraps [106], or as coating for plastic and bioplastic to confer hydrophobicity. Biodegradable polyester film has been prepared from aleuritic acid by melt-polycondensation in air. The film showed good water barrier properties and biocompatibility [107]. Similarly, films obtained by non-catalyzed melt-polycondensation of three types of tomato pomace by-products demonstrated high hydrophobicity. Furthermore, all these studies indicate that cutin has a valuable potential for packaging applications.

## 4. Liquid Bio-Based Coatings

One of the main methodologies used in the coating of cellulosic and plastic substrates is represented by the application of a liquid suspension/solution of functional molecules. 

Among liquid application techniques, the most used are mentioned in Table 2, summarizing the description and main results of spray drying, electrospray, airbrush spraying, spin coating, dipping, solution casting, flexography and gravure roll coating.

Each method can be considered a valid technique for wet coating application and the specific choice depends on the physico-chemical features of the coating and the surface properties of the chosen substrate. For instance, coatings based on polysaccharides or proteins exhibit a considerably polar component in terms of surface energy, while the cutin, composed of ω-hydroxy acids, forms hydrophobic films [135]. Similarly, the surface energy of fossil-based plastics, such as polyolefins, showed a high dispersive component [136,137], bioplastics, such as polyesters, displayed a progressive increase in the polar component [138], whereas polysaccharides showed a predominance of the polar component [139]. It was reported in the literature that good adhesion between coating and substrate strongly depends on the interfacial surface energy and the topography/geometry of the adherent bodies [140]. As the wet coating was applied through the use of a liquid it was necessary for optimal conditions to be established in the substrate and the coating solution/suspension. Commonly the evaluation of surface energy of a liquid on a solid surface is defined by the contact angle expressed by the Young’s equation and the relative work of adhesion expressed by Dupré’s equation [141,142]. Surface energy of the liquid depends not only on the selected coating but also on the chosen solvent and the presence of surfactants [143,144,145]. Instead, factors such as concentration [146,147], viscosity [148,149], and wettability also influence the homogeneity of the coating, the drying speed, and the choice of application method. Instead, factors as the concentration and viscosity, in addition to the wettability, also influence the homogeneity of the coating, the drying speed and the choice of application method. Regarding the morphology, as the liquid coating assumes the shape of the solid, it was important to evaluate the roughness and the absorbency/porosity of the substrate. In literature it was reported that roughness has a strong influence on the wettability of the surface showing lower values of contact angle at higher levels of roughness [150,151,152]. The presence of porous or high-absorbency substrates highly influences the coating process by increasing the wettability and changing the drying kinetic [153,154,155,156,157]. Although surface roughness and porosity can increase the wettability of a surface, they have a significant influence on the coating morphology and thickness uniformity [158,159]. Other aspects that influence the coating are the process parameters, such as the deposition rate [160], the drying temperature [161] and the use of air or vacuum drying [162,163]. 

Application of coating with a wet technique had some advantages that were suitable for increasing the development of bio-based coatings. The use of a room temperature application avoids the thermal degradation and hydrolysis of bio-based materials, which are inherently sensitive to these processes [164,165,166]. Moreover, the use of a liquid medium allows the wettability of this type of coating to be tuned. For instance, a concentrated coating can be more suitable for blade or dipping application than a diluted one, which conversely can be more suitable for spray application. Particular attention must be paid to the choice of solvent/suspending agent, favouring bio-based and non-toxic liquids. The use of non-toxic substances for humans and environment should be deeply investigated because it could interfere with processes such as biodegradation [167,168,169]. Unfortunately, the preparation of optimal solution and dispersion for coating could not be easy to achieve. Solution guarantees a homogeneous distribution of the coating layer in wet medium, but the coatings are strongly influenced by properties like viscosity and possible formation of gel structures [170]. Dispersion has a weak influence on the physical properties of the coatings but they request a stabilization. In particular, with the increasing availability of nanometric biomolecules, such as the CNs [75,171] or the cellulose nanowhiskers [119], these problems were amplified due to the increase in the surface area. Consequently, high energetic dispersion and homogenization techniques, such as the ultraturrax homogenization [172,173], sonication [174,175] and high pressure homogenization (HPH) [176,177], were increasingly applied. If the operative parameters and the homogenization techniques did not allow an optimal wet coating to be prepared, the use of biosurfactant [178,179] or a bio-based primer [180,181,182] becomes necessary.

## 5. Solid Functional Bio-Based Coatings

In recent years, solid coatings have been developed in an exponential way and the necessities of functional coatings have also gradually been fortified [183]. As described in Table 3, among the widely used solid coating application techniques, the most common are: co-extrusion, compression molding, fluidized bed dipping, electrostatic spray and roll coating.

A differentiation can be made between hot melt coatings (HMCs) and powder coatings. HMCs have been in use since the fifties, they relies on thermoplastic solid materials achieved without the use of solvents, which are inherently solid below 80 °C and they become low-viscosity fluids at higher [184,185]. 

HMC is made of thermoplastic materials that can be easily spread upon heating. When the hot melt is in a fluid state, it flows onto the substrate. When the hot melt is then cooled, the coating solidifies and forms a bond to the substrate [186].

Today, HMCs are involved in the production of items in many manufacturing fields, from packaging to paper industry, and their development is increasing considering the step ahead made in the hot melt coating application methods [187].

**Table 3:** Description of solid coatings application techniques and main results on solid coatings.

	Solid Coatings	
Technique	Description	Meaningful applications with solid coatings
Co-extrusion	Co-extrusion is a process that allows the simultaneous extrusion of two or more materials along the same production line, resulting in a multilayer final product [188].	[189]
Compression molding	A method based on the application of a pressure on a powder or another solid placed on a substrate in the lower plate of the press. The equipment is heated guaranteing a good adhesion between the layers [190].	[191]
Fluidized bed dipping	A powder is transformed in an entirely consolidated film thanks to electrostatic forces [192].	[193]
Electrostatic Spray	The coating method is characterized by the deposition of the solid coating through electrostatic atomization [194].	[195]
Roll-to-Roll Coating	The coating or printing process is performed spreading a solid coating on a moving substrate, constitued above all by thin and flexible polymers, papers, ot textiles [196].	[197]

As they form a strong bond quickly, simply by cooling, they are compatible with many materials Achanta et al. [198] stated that HMC methods of applications are very attractive in all sectors in which there is a fundamental necessity to develop novel, simple, efficient, precise, and cost-effective coating processes. 

The driving force for the employment of HMCs (and their strength compared to water-based film-coating technology) is to avoid the use of hazardous and toxic solvents as described by several literature works [199,200].

On this premise, since there is no necessity for solvent evaporation, the time for the process to be completed is shorter; consequently are eliminated all solvent disposal/treatment associated with organic solvents., making HMC environment-friendly materials [183].

Although water-based coating systems are useful, they are not completely flawless. A difficult problem encountered with waterborne coating systems is the variation in the dispersion of the coating. In fact, it is virtually impossible to control the presence or growth of microbes in coating dispersions without damage [201]. In addition, HMCs offer significant technical advantages, i.e., faster and cheaper coating processes and less risk of dissolution of biomolecules during treatment [202].

However, although this technique has been described well by many review papers, like by Lopes et al. [203], its application is scarce in producing coatings out of the pharmaceutical sector. The motivation is the necessity to mix in the correct way “active molecules”, able to guarantee the achievement of the desired HMC properties, with oligomers, which act as primers during a low-temperature extrusion process (to ensure that the hot melt has the right melt strength to be processed) [204].

Improving the solubility of water-insoluble molecules remains a real challenge in the development of HMC formulations, as the bioavailability of active ingredients is controlled by their solubility in water [205]. Improving the solubility of the couple “active molecule–oligomer” is one of the challenges nowadays.

Finally, it is possible to conclude that HMCs represent the best strategy to develop coatings for bioplastics and cellulose with highly diffused industrial technologies, such as extrusion coating, in which the adhesion of the coating to the bioplastic substrate is very critical, as pointed out by Correlo et al. [206].

Another solid coating can be achieved in the form of a powder. In fact, with environmental regulations becoming more stringent, an urgent problem is to reduce the use of volatile organic compounds (VOCs). An approach based on powder coatings, which is inherently solvent free is perfect from this point of view. Such coatings represent the final destination along the road to VOC reduction [207]. 

Because of their superior application properties and environmental friendliness, the use of powder coatings has grown very rapidly in recent years and the demand for functional powder coatings has gradually intensified. The components of powder coatings are extruded, crushed and screened to obtain powder for coating [208]. Powder coatings are usually operated first by electrostatic spraying and fluidised bed impregnation methods. Then, the powder is heated until it melts and hardens.

The most commonly used method for thermoplastic systems is the fluidised bed process. Here, a hot metal test piece is immersed in the fluidised powder. The powder dissolves, melts and cures, resulting in a smooth polymer surface on the test piece [209]. Due to the partial crystallisation of polyester resin, the effect on the properties of the powder coating film, especially the mechanical properties, cannot be ignored in industrial applications [210].

The production of a polymeric powder coating by extrusion is, actually, a multi-step process. Indeed, it can be labelled a “batch process” because it involves weighing, premixing, extrusion and milling, weighting the “ingredients” in prescribed ratios, pre-mixing them in the solid state, feeding them into an extruder so to obtain a molten homogenous mixture. The molten material, after cooling, is subsequently crushed into flakes of about 10–20 mm and then finally ground by disc or hammer mill to obtain particles with size in the range of 2–100 μm with a distribution peak of about 50 μm [211]. 

Powder coating formulations exist on the market either as thermosetting or thermoplastic but they are fossil-based. Concerning biopolyester thermoplastic-based powder coatings there are still many steps ahead to reach an industrial application. Interestingly, Van Haverman et al. [212] developed alkyd resins for high-solid powder coatings completely based on commercially available renewable resources.

As interest continues to focus on improving more sustainable technologies, and as the prices of fossil raw materials are set to rise, the coming decades will inevitably see an increase in renewable-based coatings, combining them with unique properties.

## 6. Future Perspectives for Liquid and Solid Bio-Based Coatings

The present review evidenced the needs of formulating new bio-based coatings, which can be highly compatible with cellulosic and bioplastic substrates, in which thermoplastic starch films are one of the main examples [213]. The use of proper food or agricultural waste for their formulation agrees with the circular economy principles, can keep the cost of new materials down and can result in evident environmental advantages.

It is easy to predict, on the basis of the present literature survey, that chitin/chitosan coatings could be interesting both in liquid and solid forms. Cellulosic substrates [26], but also bioplastic [214] and textile substrates [113], can be easily treated with liquid coatings. The penetration of the liquid in the cellulosic or textile tissue is an important aspect to be controlled. Whereas chitosan, dissolved in acidic water, can penetrate inside the tissues, the chitin nanofibrils, generally suspended in water, remain on the substrate surface. In both cases the antimicrobial action can be modulated by controlling the concentration of these biopolymers in the liquid product. Solid coatings in powder or in film can be highly innovative. CNs or chitosan could be properly dispersed in thermoplastic matrices, having a low melting temperature for an easy and not expensive coating in terms of energy application.

Proteins can actually be used more on plastic and cellulosic substrates for developing high oxygen barrier coating for plastic and cellulose packaging [81,93], but they could also be potentially employed in solid coating formulations, despite their difficult processability and temperature sensitivity [215].

A cutin lacquer was developed for metallic substrates [103], but it is potentially applicable by liquid coatings on cellulosic and bioplastic substrates to obtain coloured (i.e., not transparent) coatings. The high hydrorepellency of cutin could probably allow these properties to be modulated on many substrates. The application of cutin in solid coating would be very new and interesting for the same reason.

These last considerations are summarized in Table 4. 

In general, the preparation of liquid coatings based on chitin/chitosan, protein or cutin is at a higher technological readiness level, with respect to solid coatings. 

The latter are extremely promising but more challenging than liquid coatings, as the modulation of morphological features based on coating concentration is a complex issue, as well as for the possible thermal degradation that could occur during processing and further application.

**Figure 2:**
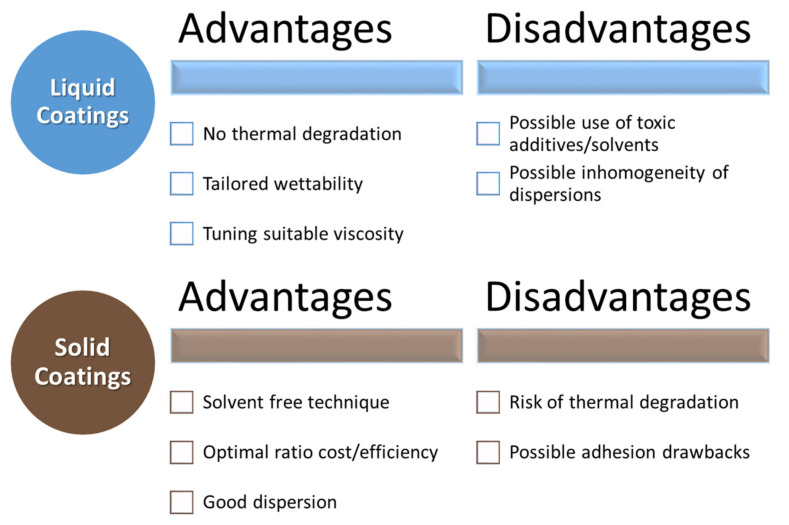
Advantages and disadvantages of liquid and solid coatings.

The considered biopolymers are thus extremely promising for developing innovative and environmentally friendly coatings for several substrates with some pros and cons, shown in Figure 2. These coatings can be extremely useful for improving the properties of renewable products, thus boosting their use in several applications.

## 7. Conclusions

The objective of this review has been to summarize the main techniques for the application of bio-based coatings, differentiating between liquid and solid methods. Moreover, an in-depth literature search was necessary to evaluate some properties, which can be obtained starting from the dispersion of biomolecules within the coating itself. Chitosan/chitin, proteins and cutin were the main focus of this review paper, because of their complementary functional properties, antimicrobial, oxygen and water barrier, respectively. These properties are highly requested in novel functional bio-based coatings. Liquid and solid bio-based coatings showed advantages and disadvantages, but they can provide high flexibility to industry as well as drive specific innovations in the market, thus satisfying the exigencies of more sustainable yet performant products, than fossil-based counterparts.

In conclusion, this paper evidenced that the world of bio-based coatings is constantly evolving and expanding; several sectors are looking for a bio-based solution to improve the properties of their substrates and a considerable technological step forward has been made in this field. 

## Figures and Tables

**Table 1:** Brief overview of biomolecules mostly used for coating formulations.

Bio-Based Polymer	Preparation	Application Method	Properties Improved and Main Results	REF
Chitin	0–2 wt.% chtin nanowhiskers dissolved in H_2_SO_4_ and glycerol.	Casting method on maize-starch films.	Evident antimicrobial resistance vs. Gram-positive *Listeria monocytogenes.*	[30]
	2 wt.% of water suspension of nanofibrils dispersed in PEG 8000.	Spray dryer on bioplastics films.	Antimicrobial and skin-regenerative improvements.	[31]
Chitosan	Chitosan (2 wt.%) and glycerol (2 wt.%) dissolved in a 1% (vol/vol) aqueous solution of acetic acid.	Chromatography plate coater application onto PP films. corona-treated	Evident antimicrobial resistance vs. *Listeria monocytogenes*, *Staphylococcus aureus*, and *Escherichia coli.*	[32]
	Chitosan concentration of 0.02 g/mL in acetic acid mixed in equal volumes with hydroxypropyl methylcellulose.	Thin-layer chromatography plate coater on plastic films.	Excellent long-term antilisterial effect.	[33]
Lignin	Dissolution in acetone of different amounts of softwood kraft lignin and evaporation of the solvent.	Erichsen coater on to a paperboard substrate.	Evident decrease in Oxygen Transmission Rate (OTR) value and a stable contact angle with respect to paperboard alone.	[34]
	Lignin estereified with palitic and lauric acid chloride in a mixture 3:1 ethanol/water.	Erichsen coater on a commercial paperboard substrate.	Good barrier properties against O_2_ and H_2_O	[35]
Cellulose derivates	Cellulose nitrate ester (CMCN) were dissolved in mixed solvents systems in different amounts.	Solvent casting method.	Gas and water barrier optimized.	[36]
	Hydroxypropyl methylcellulose acetate succinate plasticized with triethyl citrate and acetylated monoglyceride	Centrifugal granulator for feeding the coating powder and spraying simultaneously the plasticized.	Improved gastric resistance, coating efficiency, and processing stability	[37]
Proteins	Whey proteins with hydrolysed lactose at different contents	“Bird-type” applicator onto paperboard substrates	Good grease resistance and minimization of plasticizer migration	[38]
	12 g of whey proteins in 6 g of glycerol and 30 g of deionized water	Compression molding onto cellulosic substrates	Gas-barrier properties improvements	[39]

**Table 2:** Liquid coatings techniques and main results regarding liquid bio-based coatings.

LIQUID COATINGS		
Technique	Description	Meaningful Applications in Liquid Bio-Based Coatings
Spray Drying	Transformation of a solution in which are dispersed particles into dried ones, thanks to a gaseous hot drying medium [108].	[109,110,111]
Electrospray	Liquid atomization through commanding electrical forces on the flow of a liquid injection from a cilindric die. This technique gaurantees uniform droplets generation [112].	[113,114]
Airbrush Spraying	Polymer solutions are sprayed through an airbrush supplied by a nitrogen line and fixed on a mechanic arm over a hot plate [115].	[116,117]
Spin Coating	The material used to coat is present at the centre of the substrate, then it is rotated at high speed until centrifugal force spreads the coating material [118].	[119,120]
Dipping	The solution substrate is immersed in the coating for effective formation of the complete material [121].	[122,123]
Solution Casting	A polymer is dissolved in a solution into which an inner diameter mold is immersed. The solvent is removed to leave a solid cast layer. This layer can be laminated or coated before being stripped from the mold [124].	[125,126,127]
Flexography	Flexographic assumes the possibility to widespread liquid inks with a low viscosity on paper, cardboard, or plastic films [128].	[129,130]
Gravure Roll Coater	Coating is introduced onto the surface of an engraved roll, then it is partially submersed in or by an enclosed applicator head that holds the coating against the roll [131].	[132,133,134]

**Table 4:** Predictable perspectives for chitin/chitosan, protein and cutin on different substrates.

Biomolecule	Liquid	Solid
Chitin/chitosan	Antimicrobial coatings for cellulose, bioplastic and textile substrates.	Potentially antimicrobial and water barrier coatings for cellulose, plastic and textile substrates.
Protein	High oxygen barrier coatings for plastic and cellulose.	In blend with polyesters, oxygen barrier coatings for cellulose and plastic.
Cutin	Hydrorepellent coatings and potentially for cellulose, bioplastic, and textile substrates.	Potentially hydrorepellent coatings for cellulose, bioplastic, and textile substrates.
